# Diagnostic dilemma in diagnosing rare cause of protein losing enteropathy: Waldmann's disease

**DOI:** 10.1002/ccr3.5992

**Published:** 2022-06-21

**Authors:** Sarita Prajapati, Sujan Bohara, Gaurab Mainali, Samikshya Karki, Sharan Thapa, Nirjala Aryal

**Affiliations:** ^1^ Department of Pediatrics Birendra Military Hospital Kathmandu Nepal; ^2^ Department of General and Gastrointestinal Surgery Nepal Mediciti Hospital Lalitpur Nepal; ^3^ Nepalese Army Institute of Health Sciences Kathmandu Nepal; ^4^ Lekhnath City Hospital Private Limited Pokhara Nepal

**Keywords:** intestinal lymphangiectasia, medium‐chain triglycerides, protein‐losing enteropathy, Waldmann's disease

## Abstract

Waldmann's disease, or primary intestinal lymphangiectasia, is an unusual cause of protein‐losing enteropathy primarily characterized by lymphopenia, hypoalbuminemia, and hypogammaglobulinemia. However, variable clinical presentations result dilemmas in diagnosis and effective management. We present a toddler diagnosed with Waldmann's disease managed with a high‐protein diet and medium‐chain triglyceride supplementation.

## Graphical abstract

Waldmann's disease, or primary intestinal lymphangiectasia, is an unusual cause of protein‐losing enteropathy primarily characterized by lymphopenia, hypoalbuminemia, and hypogammaglobulinemia. However, variable clinical presentations result in dilemmas in diagnosis and effective management.

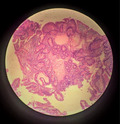

## 1 INTRODUCTION

Intestinal lymphangiectasis is a rare cause of protein‐losing enteropathy characterized by diffuse or local dilatation of the enteric lymphatics, mostly presenting with features of hypoproteinemia leading to bilateral lower limb edema, ascites, or rarely anasarca. Waldmann and Schwabb in 1961 reported this rare disease with intestinal protein loss leading to hypoproteinemia and anasarca.^1^ Intestinal lymphangiectasia can be either primary (idiopathic) or secondary. Primary intestinal lymphangiectasia (PIL) usually occurs in children and adolescents due to the congenital deformity of the small bowel lymphatic system, whereas secondary intestinal lymphangiectasia is more often seen in adults and occurs secondary to elevated lymphatic pressure such as in lymphoma, systemic lupus erythematosus, inflammatory bowel disease, malignancies, constrictive pericarditis, and cardiac surgery.^2^

Primary intestinal lymphangiectasia is a congenital lymphatic system disorder characterized by marked ectasia of lymphatic vessels, resulting in lymph fluid obstruction and leakage.^3^ With different presentations, the time of presentation may vary from early childhood to adulthood. Histopathological examination of biopsy taken from the involved part of the intestine demonstrates dilated lymphatics in the mucosa, submucosa, and serosa in the absence of coexisting inflammation, which is used to diagnose primary intestinal lymphangiectasia.^2^, ^4^, ^5^ Treatment options range from symptomatic measures such as nutritional supplements to advanced surgical procedures.^2^, ^3^

We herein reported a three‐year‐old toddler with primary intestinal lymphangiectasia who presented with periorbital edema, abdominal distension, and bilateral pitting edema, that is, generalized anasarca, which was managed with medium‐chain triglycerides, a high protein diet, and a multivitamin with fat‐soluble vitamin supplementation.

## 2 CASE PRESENTATION

A three‐year‐old female toddler, born of a non‐consanguineous marriage, presented to our pediatric department with complaints of abdominal distension since 1 month and periorbital and bilateral limb swelling since 3 days. There was no history of passing red‐colored urine, urgency, frequency, painful micturition, cough, altered sensorium, abnormal body movement, headache, photophobia, rash, or joint pain. Her family had no history of recurring infections, chronic illnesses, or significant genetic diseases. Her feeding and activities were normal.

Her anthropometric and nutritional evaluations were confirmed as follows: Height‐91 cm, Weight‐14.5 kg, Head circumference (HC)‐47 cm, Mid‐Upper Arm Circumference (MUAC)‐13 cm, Weight for age—0 to −1 SD, Height for age—0 to +1 SD (Standard deviation), Weight for height— +1 to +2 SD.

Physical examination revealed bilateral lower limb pitting edema with periorbital edema and a distended abdomen (with an abdominal girth of 56 cm at umbilicus level), flank fullness, a slit‐like centrally placed umbilicus, fluid thrill, and shifting dullness, but no hepatosplenomegaly. All other systemic findings and vital parameters were normal.

**FIGURE 1:**
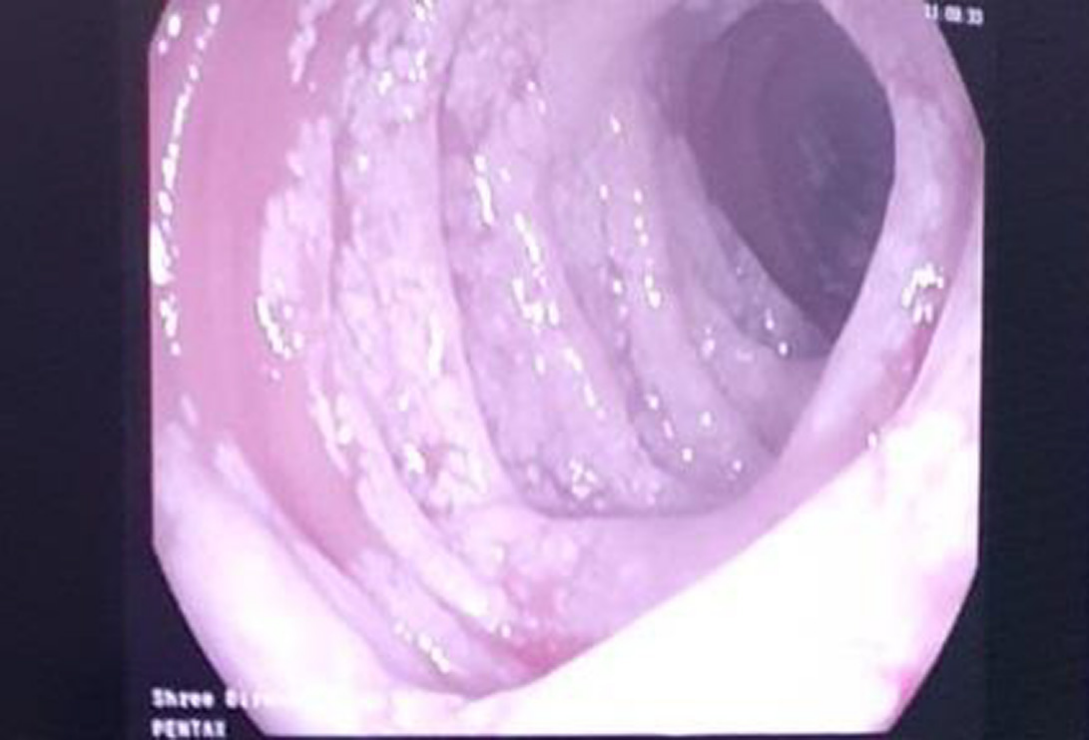
Enteroscopy showing white streaks or patches over second part of duodenum

**FIGURE 2:**
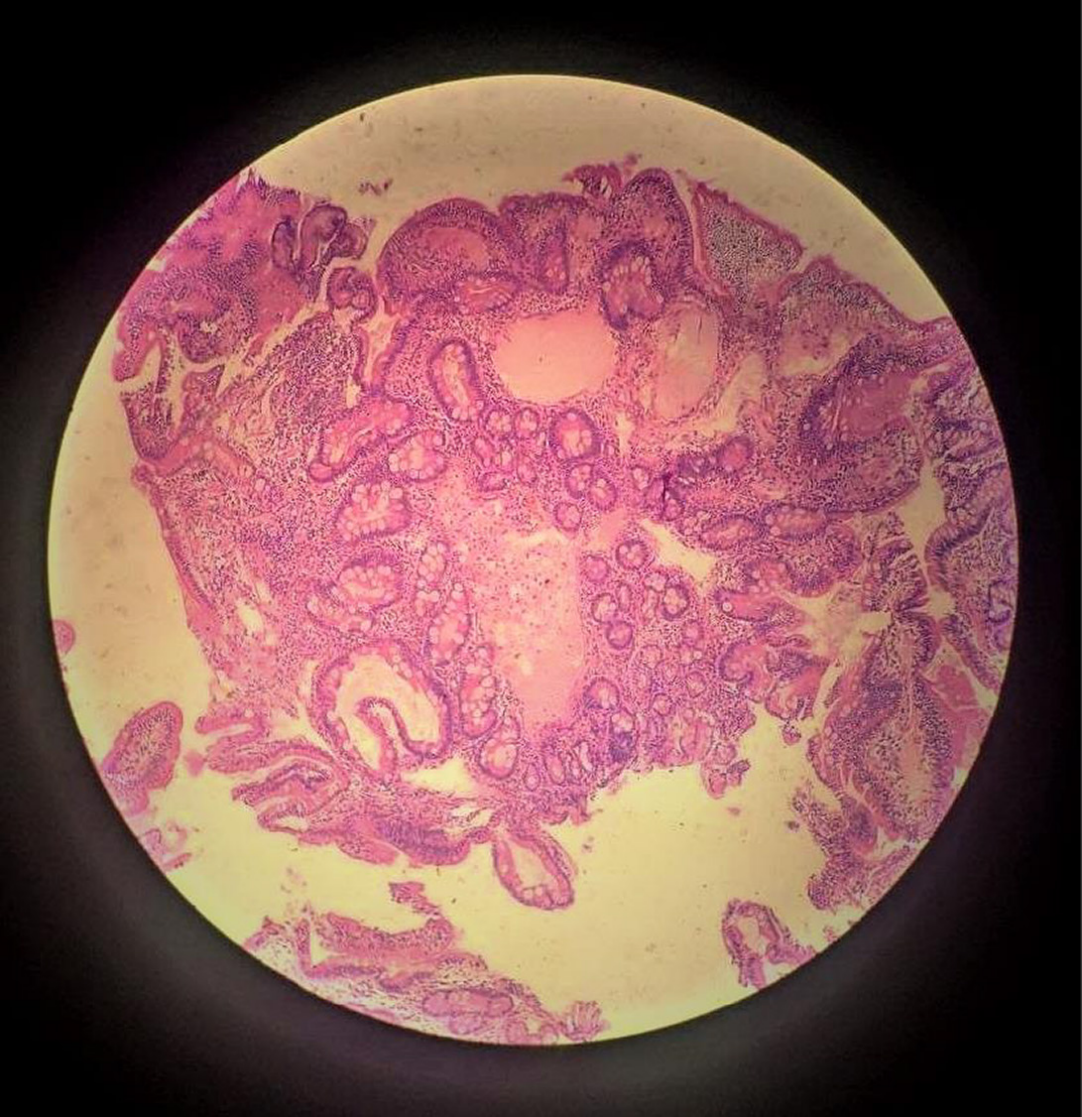
Hematoxylin and Eosin stain of duodenal biopsy showing columnar lining epithelium with apical goblet cells along with multiple dilated lymphatic channels in lamina propria suggestive of intestinal lymphangiectasia

The patient had been admitted with a provisional diagnosis of nephrotic syndrome and further work‐up was done. Initial laboratory reports revealed anemia (Hemoglobin 11.2 gm/dl), lymphopenia (15%), thrombocytosis (668,000/mm^3^), hypocalcaemia (8 mg/dl), normal C‐reactive protein, liver function test, renal function test, electrolytes, lipid profile, and thyroid function test. Peripheral blood smears showed microcytic hypochromic anemia with thrombocytosis. The iron profile and fat soluble vitamin assessment showed a decreased total iron level (54 mcg/dl) and decreased vitamin D (13.6 ng/dl), respectively. Ultrasonography of the whole abdomen showed moderate ascites, multiple enlarged mesenteric lymph nodes with mildly diffused mucosal thickening of the bowel loop but normal shape, outline, and echo‐texture of the hepatic, renal, and splenic structures. Urinalysis, culture and sensitivity, and a 24‐h urinary protein (2.7 gm/dl) were normal, which ruled out the renal cause. Transthoracic echocardiography appeared normal, thus ruling out cardiac causes through clinical examination and echocardiography. Ascitic fluid analysis revealed triglyceride‐rich chylous ascites (exudative type). Upper gastrointestinal endoscopy (Figure 1) showed normal mucosa over the esophago‐gastric region along with white patches or streaks over the 1st and 2nd parts of the duodenum. The duodenum was stained with H and E (Figure 2) to reveal tissue lined by columnar epithelium forming crypts and villi with apical goblet cells and multiple dilated lymphatic channels in the lamina propria with inflammatory infiltrates of lymphocytes, plasma cells, and eosinophils, indicating intestinal lymphangiectasia. Colonoscopy showed no distinguishable mucosal abnormalities. Other biological tests like parasitological stool examination, serological tests (Human Immunodeficiency Virus, Cytomegalovirus), and tuberculosis assessment appear normal. Anti‐nuclear Antibody (ANA), anti‐double stranded DNA, and IgA, anti‐tissue transglutaminase, were negative. Serum electrophoresis revealed a decrease in IgG serum protein with normal other types of proteins.

Considering all the clinical history examination and investigations, a diagnosis of primary intestinal lymphangiectasia (Waldmann's Disease) was made. Then, the child was treated with human albumin infusion as a protein supplement (at 1 gm/kg, 75 ml over 3 h once a day for 3 days) with furosemide, multivitamins including fat‐soluble vitamins, oral iron (syrup Ferro folic at 4 mg/kg/day), calcium supplementation (syrup calvit at 40 mg/kg/day), and MCT (Medium Chain Triglyceride)‐based oils. She responded dramatically to the treatment, with gradual stabilization and improvement in her clinical conditions, and was discharged on the third week following her admission with advice on the consumption of a protein‐rich diet, a low‐fat diet, and MCT oil‐based food supplementation on her diet as a lifelong dietary therapy.

Regular follow‐up was done once a month for the first 3 months and then every 3 months from the diagnosis till 18 months after the diagnosis. During the follow‐up period, no symptoms developed, and the investigation was within normal limits.

## 3 DISCUSSION

Waldmann et al. first described 18 cases of “idiopathic hypercatabolic hypoproteinemia” in 1961.^1^ Intestinal lymphangiectasia is an uncommon but important cause of protein loss intolerance and is characterized by poor lymph drainage in the small intestines associated with enlarged lymphatic channels. The prevalence of clinically relevant PIL is not known.^2^, ^6^ It occurs mainly in children and is equally common among both sexes.^6^, ^7^ Although most cases are sporadic, Le Bougeant et al. reported rare familial forms of Waldmann's disease.^8^, ^9^

Primary and secondary are the two types of intestinal lymphangiectasia described in the literature according to the cause of the disease. In primary lymphangiectasia, there is no predisposing condition to increased lymph pressure and it is probably caused by a congenital anatomical malformation of the lymphatic system. PIL is associated with Yellow Nail Syndrome, Klippel‐Trenaunay‐Weber syndrome, Von Recklinghausen, Noonan, Turner, and Hennekam.^1^, ^2^, ^3^, ^4^ In our case, there is no known associated disease. Hokari et al. reported inconsistently changed expressions of regulatory molecules for lymphangiogenesis in the duodenal mucosa of PIL patients.^10^ Secondary intestinal lymphangiectasia is an acquired disease characterized by increased leakage of the intestinal lymph, secondary to an increase in pressure due to obstruction in the lymphatics.^2^

The diagnosis is based on a set of clinical, biological, radiological, endoscopic, and histopathological criteria. The disease is characterized by hypoproteinemia, lymphocytopenia, edema, and ascites. Clinical manifestations may be presented as asymptomatic or fatigue, lower abdominal pain, edema, chylothorax, chronic diarrhea, and ascites.^1^, ^2^, ^3^, ^4^, ^5^, ^6^, ^8^ Iron deficiency anemia and hypocalcemic tetany can be seen.^2^, ^8^ In addition, our case had iron and calcium/Vitamin D3 micronutrient deficiencies. Along with the above clinical features, stool analysis may show steatorrhea and increased alpha‐antitrypsin clearance (>24 ml/day).^2^, ^8^, ^11^ The presence of intestinal lymphangiectasia is confirmed by the presence of intestinal lymphangiectasia based on endoscopic findings and the corresponding histology of intestinal biopsy specimens.^12^ Histological examination of duodenum‐jejunum and ileum biopsies confirms the presence of lacteal juice, dilated mucosal (from moderate to severe) and sub‐mucosal lymphatic vessels (and also in the serosa) with polyclonal normal plasma cells.^2^, ^11^ Martins et al. reported an enteroscopy showing white spots with a “snowflake” appearance, which are the typical findings of intestinal lymphangiectasia.^8^ Sometimes, endoscopy may be negative when there is a segmental or localized lesion. In those cases, videocapsule endoscopy is a useful tool to detect the presence of intestinal lymphangiectasia and to specify its localization.^2^ Exclusion of secondary causes of intestinal lymphangiectasia like erosive and non‐erosive intestinal disorders, conditions involving mesenteric lymphatic obstruction, and cardiovascular disorders that increase central venous pressure is necessary for a definitive diagnosis of Waldmann's disease.^8^

A wide range of presenting symptoms provides a challenge to appropriate therapy. The treatment protocol depends on the presenting symptoms, localization of the disease, and associated complications. The treatment is based on a diet free of long‐chain lipids, enriched with protein and medium‐chain triglycerides.^2^, ^3^, ^4^, ^5^, ^6^, ^7^, ^8^, ^11^ But, Ballinger et al. stated that it does not always give good results..^13^ In literature reported by Aoyagi et al., in patients not responding to a low‐fat diet, enteral nutritional therapy (elemental, semi‐elemental, and polymeric diets) may be required.^14^ It was reported that somatostatins such as octreotide can decrease triglyceride absorption.^2^, ^3^, ^8^, ^13^ Antiplasmin therapy may have some role when fibrinolysis is increased.^2^, ^11^ Surgical treatment is helpful for localized intestinal involvement or intestinal obstruction.^2^, ^8^

## 4 CONCLUSIONS

Rarity and variable presentation of disease can led into dilemma in diagnosing the disease and management. Clinician should be aware about this rare disease and consider as one of the differential whenever patient clinically presents with anasarca, lymphedema with peri‐orbital puffiness. Though rare, it can be managed early and effectively with dietary therapy and nutritional supplementation.

## AUTHORS CONTRIBUTIONS

SP contributed to the collection of the case information; SP, SB, GM, SK, and ST contributed to reviewing the literature and designing and writing the manuscript. SB, SK, and GM also contributed to revising, re‐editing, and reshaping the manuscript. NA established the diagnosis and revised the manuscript critically for important intellectual content. All authors read and approved the final version of the manuscript.

## CONFLICT OF INTEREST

None.

## ETHICAL APPROVAL

Study did not include experiments on humans or animals.

## CONSENT

For the publication of this case report, informed written consent was taken from the patient's parents.

## Data Availability

None.
